# Molecular epidemiology of *Streptococcus agalactiae* in non-pregnant populations: a systematic review

**DOI:** 10.1099/mgen.0.001140

**Published:** 2023-11-29

**Authors:** Luria Leslie Founou, Uzma Basit Khan, Nubwa Medugu, Tatiana C. A. Pinto, Saffiatou Darboe, Zhu Chendi, Raspail Carrel Founou, Ka-Ning To, Dorota Jamrozy, Konstantinos Karampatsas, Victoria R. Carr, Kevin Pepper, Ziyaad Dangor, Margaret Ip, Kirsty Le Doare, Stephen D. Bentley

**Affiliations:** ^1^​ Reproductive, Maternal, Newborn and Child Health (ReMARCH) Research Unit, Centre of Expertise and Biological Diagnostic of Cameroon Research Institute (CEDBCAM-RI), Yaoundé, Cameroon; ^2^​ Bioinformatics and Applied Machine Learning Research Unit, EDEN Biosciences Research Institute (EBRI), EDEN Foundation, Yaoundé, Cameroon; ^3^​ Antimicrobial Research Unit, School of Health Sciences, University of KwaZulu-Natal, Westville Campus, Durban, 4041, South Africa; ^4^​ Parasites and Microbes, Wellcome Sanger Institute, Wellcome Genome Campus, Hinxton CB10 1SA, UK; ^5^​ Department of Medical Microbiology and Parasitology, National Hospital Abuja, Abuja, Nigeria; ^6^​ Instituto de Microbiologia Paulo de Goes, Universidade Federal do Rio de Janeiro, Rio de Janeiro, Brazil; ^7^​ Medical Research Council Unit at London School of Hygiene and Tropical Medicine, Banjul, Gambia; ^8^​ Department of Microbiology, the Chinese University of Hong Kong, Hong Kong, PR China; ^9^​ Department of Microbiology, Hematology and Immunology, Faculty of Medicine and Pharmaceutical Sciences, University of Dschang, Dschang, Cameroon; ^10^​ Antibiotic Resistance Infectious Diseases (ARID) Research Unit, Centre of Expertise and Biological Diagnostic of Cameroon Research Institute (CEDBCAM-RI), Yaoundé, Cameroon; ^11^​ Institute of Infection and Immunity, St George’s University of London, London, UK; ^12^​ Centre for Host–Microbiome Interactions, Faculty of Dentistry, Oral and Craniofacial Sciences, King’s College London, London, SE1 9RT, UK; ^13^​ Vaccines and Infectious Diseases Analytics (VIDA) Research Unit, University of the Witwatersrand, Johannesburg, South Africa; ^14^​ Medical Research Council Uganda Virus Research Institute and London School of Hygiene and Tropical Medicine Uganda Research Unit, Entebbe, Uganda; ^15^​ Department of Pathology, University of Cambridge, Cambridge, UK

**Keywords:** antibiotic resistance, molecular epidemiology, non-pregnant populations, One Health, *Streptococcus agalactiae*, virulence

## Abstract

*
Streptococcus agalactiae* (group B *Streptococcus *, GBS) has recently emerged as an important pathogen among adults. However, it is overlooked in this population, with all global efforts being directed towards its containment among pregnant women and neonates. This systematic review assessed the molecular epidemiology and compared how the lineages circulating among non-pregnant populations relate to those of pregnant and neonatal populations worldwide. A systematic search was performed across nine databases from 1 January 2000 up to and including 20 September 2021, with no language restrictions. The Joanna Briggs Institute (JBI) Prevalence Critical Appraisal Tool (PCAT) was used to assess the quality of included studies. The global population structure of GBS from the non-pregnant population was analysed using *in silico* typing and phylogenetic reconstruction tools. Twenty-four articles out of 13 509 retrieved across 9 databases were eligible. Most studies were conducted in the World Health Organization European region (12/24, 50 %), followed by the Western Pacific region (6/24, 25 %) and the Americas region (6/24, 25 %). Serotype V (23%, 2310/10240) and clonal complex (CC) 1 (29 %, 2157/7470) were the most frequent serotype and CC, respectively. The pilus island PI1 : PI2A combination (29 %, 3931/13751) was the most prevalent surface protein gene, while the tetracycline resistance *tet*M (55 %, 5892/10624) was the leading antibiotic resistance gene. This study highlights that, given the common serotype distribution identified among non-pregnant populations (V, III, Ia, Ib, II and IV), vaccines including these six serotypes will provide broad coverage. The study indicates advanced molecular epidemiology studies, especially in resource-constrained settings for evidence-based decisions. Finally, the study shows that considering all at-risk populations in an inclusive approach is essential to ensure the sustainable containment of GBS.

## Data Summary

The authors confirm that all supporting data, code and protocols have been provided within the article or through supplementary data files.

**Impact Statement**

*
Streptococcus agalactiae* (group B Streptococcus, GBS) is an important yet often overlooked micro-organism that can cause illness in newborns, infants and pregnant women, as well as adolescent or non-pregnant adults. One of the main challenges in recognizing GBS in non-pregnant individuals as a serious public health threat is the poor understanding on how GBS infections or colonization in non-pregnant individuals might affect pregnant women and newborns around the world. In this study, we retrieved papers studying GBS in non-pregnant populations and exploited existing genomics data for GBS isolates to investigate how GBS spreads among non-pregnant people and how it can relate to GBS colonizing or causing infections in pregnant women and babies. The study showed that more data, especially in low- and middle-income countries, are needed to control GBS effectively and make decisions based on solid evidence. The study emphasizes that it is critical to look at all groups of people who are at risk of GBS colonization/infection in an inclusive-population approach to ensure the better prevention and containment of GBS globally.

## Introduction

*Streptococcus agalactiae* (group B *Streptococcus *, GBS) is a leading cause of neonatal infections and deaths worldwide, particularly in low and middle-income countries (LMICs) [1]. For maternal immunization by vaccine, the World Health Organization (WHO) identified GBS as a high-priority pathogen [2]; a hexavalent (serotypes Ia, Ib, II–V) vaccine is undergoing clinical trials, but there are currently no licensed GBS vaccines. There is limited information regarding GBS transmission and diversity beyond pregnancy, albeit some reports revealed that GBS colonize non-pregnant individuals at similar or higher rates than pregnant women, including fatal outcomes [3]. The major barriers to recognizing GBS in non-pregnant populations as a public health threat include sampling rarity, the subsequent scarcity of data and the lack of understanding concerning how non-pregnant populations contribute to maternal/neonatal invasive GBS disease (iGBS) globally.

To the best of our knowledge, this is the first systematic review on the molecular epidemiology of GBS in non-pregnant populations considering the phylogenetic relationship with invasive and non-invasive neonatal and pregnant women isolates. This systematic review thus aims, from a non-pregnant population perspective, to (i) determine the serotypes and genotypes of GBS isolates considering geographical variation and economic income, (ii) highlight the antibiotic resistance and surface protein genes in GBS isolates, and (iii) compare the clonal relatedness of circulating lineages among non-pregnant populations. Furthermore, pregnant women and neonates were used as comparative groups in phylogenetic analyses.

## Methods

The Preferred Reporting Items for Systematic Reviews and Meta-analyses (PRISMA) 2020 statement was followed [4] (Table S1, available in the online version of this article) (Prospero registration number CRD42021279826).

### Ethical consideration

No ethical approval was requested given that this systematic review was based solely on published reports.

### Outcome of interest

The outcome of interest was invasive and non-invasive GBS in non-pregnant populations. The non-pregnant population was defined as children (>90 days to 17 years), adults (18–64 years) and the elderly (≥65 years).

### Search strategy and study selection

A systematic search was carried out independently by nine authors (L.L.F., R.C.F., S.D., N.M., T.P., U.B.K., K.T., S.Z., M.I.) across nine databases from 1 January 2000 up to 20 September 2021, using a combination of Boolean operators (AND/OR), medical subject heading (MeSH) and pre-defined keywords (Table S2). The following nine databases were searched: Cochrane Library, African Journals Online, EBSCOhost, PubMed, Web of Knowledge, the World Health Organization Library (WHOLIS), Latin American and Caribbean Health Sciences Literature (LILACS), WANFANG MED Online and China National Knowledge.

A year restriction was applied to 1 January 2000 to ensure that the analysis focuses solely on contextual literature that depicts current trends and the genomic evolution of GBS. There were no language restrictions and the study inclusion criteria were the following : (i) original research; (ii) having at least 20 patients or 20 GBS isolates; (iii) reporting at least the serotype of GBS in neonates >3 months and/or non-pregnant adults (men, women, the elderly); (iv) reporting on the molecular characteristics of GBS, i.e. sequence type, and/or specific surface protein genes, and/or antimicrobial resistance genes, and/or virulence genes, and/or mobile genetic elements of isolated GBS in the selected populations.

The retrieved papers were independently screened for eligibility by the same nine authors based on titles and abstracts in Rayyan [5]. Thereafter, the full texts of eligible papers were assessed independently by five authors (L.L.F., U.B.K., N.M., Z.C. and T.P.) based on pre-defined inclusion and exclusion criteria (Table S3). Inconsistencies and disagreements among authors were resolved by consensus or majority. Efforts were made to contact the authors when full texts could not be retrieved and when data were missing or incomplete. A ‘hand’ search was conducted in the reference list of all selected papers. Studies originating from the same authors were considered individually if their aims were clearly different, while those with similar or related aims were considered only once.

### Full-text assessment and data extraction process

Papers were managed using EndNote (version 20, Thomson Reuters) and the data were abstracted from eligible papers independently by five authors (L.F., U.B.K., N.M., Z.C. and T.P.) using a standardized data extraction spreadsheet in Excel (Microsoft Office Excel 2016). Relevant data from papers included study details, participant characteristics, microbiological and molecular methods, and prevalence of variables of interest. The authors ascribed the WHO regions to each study. Studies where only part of the population met our inclusion criteria were included as far as it was possible to extract the data for the study participants that met our inclusion criteria.

### Qualitative assessment

The Joanna Briggs Institute (JBI) Prevalence Critical Appraisal Tool (PCAT) was used to assess the quality of included studies [6]. This tool assesses methodological quality, based on a set of nine questions that can take a ‘yes’, ‘no’, ‘unclear’ or ‘not applicable’, with a ‘yes’ scoring 1 point and ‘no’ 0 points. A score ≥7 indicates a high-quality study, a score of 4–6 implies moderate quality and a score ≤3 suggests a low-quality study. The qualitative assessment was performed independently by five authors (L.L.F., U.B.K., Z.C., S.D. and N.M.), with disagreement resolved by consensus. The same five authors independently extracted data on sample size, sample type, phenotypic and molecular methods, and GBS genomic features from included studies.

### Statistical analysis

Frequencies and percentages of serotype, clonal complex (CC), antibiotic resistance and surface protein genes were calculated in Microsoft Excel (2016) using raw data extracted from all included articles. For each study, the serotype rates were calculated by dividing the total serotype-specific isolates by the total number of GBS isolates. Descriptive statistics were also applied to calculate frequencies of CCs, resistance and surface protein genes. Data analysis was performed using R software (version 2023.03.15) and RStudio (version 2023.03.0+386). Chi-square and Fisher exact tests were used to assess the significance of the results, with a *P*-value <0.01 being considered statistically significant.

### Phylogenetic reconstruction

A phylogenetic reconstruction was implemented to assess the evolutionary relationships of isolates detected in this study and globally. The European Nucleotide Archive (https://www.ebi.ac.uk/ena/) was used to download the whole-genome sequences (WGS) of 6018 non-pregnant GBS isolates from included studies [7–11]. These data were further supplemented with 1957 neonatal invasive disease, 1519 maternal carriage and 46 maternal disease GBS genomes available online as of 30 May 2022, to assess clonal relatedness between GBS of three different populations. The quality of genomes was checked using the GBS QC pipeline v1.0.3 (https://github.com/sanger-bentley-group/GBS_QC_nf) using default settings. Serotype, sequence type (ST), antibiotic resistance and surface protein genes were called through the GBS typing pipeline v1.0.11 (https://github.com/sanger-bentley-group/GBS-Typer-sanger-nf). PHYLOViZ v2.0 and the goeBURST algorithm were used to establish relationships between STs using 3 as the minimum single-locus variant (SLV) count for subgroup definition and 6 as the minimum number of identical loci for group definition. CCs were described and subgroup analysis of CC1 isolates was undertaken to provide further evolutionary details. Sequence reads were mapped against the *S. agalactiae* strain SS1 reference genome (NZ_CP010867.1) using SMALT v0.7.4 (https://www.sanger.ac.uk/science/tools/smalt-0). Regions representing putative MGEs were masked using remove_blocks_from_aln (https://github.com/sanger-pathogens/remove_blocks_from_aln) and SNPs were identified with SNP-sites [12]. Fasttree v2.1.10 was used to construct a maximum-likelihood phylogenetic tree using snps alignment of CC1. Clusters within CC1 tree were identified using fastbaps v1.0.6 (https://github.com/gtonkinhill/fastbaps).

## Results

### Literature search and study selection

**Fig. 1.**
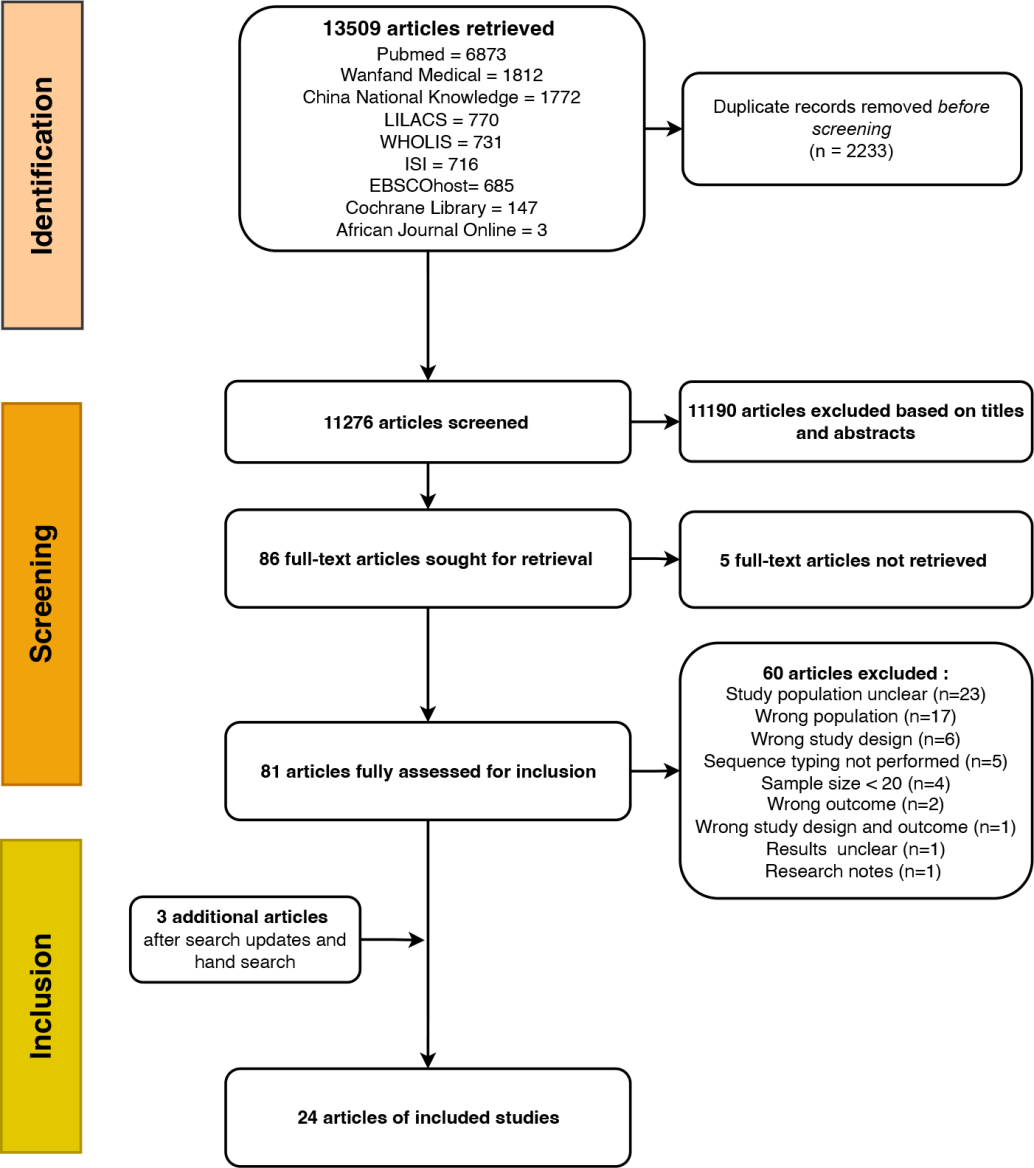
PRISMA flow chart summarizing the study selection process.

Altogether, 13 509 papers were retrieved through systematic and hand searching from the nine databases. The peer-reviewed papers retrieved were in English, French, Spanish, Portuguese and Chinese. After de-duplication, 11 276 papers were assessed for probable inclusion based on titles and abstracts. Of these, 86 papers were eligible for full-text assessment based on pre-defined inclusion and exclusion criteria, but five full-texts were not retrieved and three were added after a hand search. Two studies [8, 10] shared the same isolates with different aims but were considered as one study. Twenty-four studies were finally eligible for the analysis (Fig. 1). All studies were either high (18/24, 75 %) or moderate quality (6/24, 25 %) (Table S4). The specific characteristics of the included studies are provided in the following sections.

### Description and characteristics of studies included in the systematic review

Twelve studies were conducted across the WHO European region, mainly in the Netherlands [13, 14], Denmark [15, 16] and Sweden [17, 18]. Likewise, six studies originated from the WHO Western Pacific [19–24] and Americas regions [7–11, 25, 26] (Table 1). No study originated from the African, Eastern Mediterranean or Southeast Asian regions. Twelve studies [7, 14–16, 19, 21, 22, 25, 27–30] only investigated the genomic epidemiology of GBS in diseased adults, whereas the remaining considered GBS concomitantly in non-pregnant populations and neonates and/or pregnant women (8–11, 13, 17, 18, 20, 23, 26, 31–33; Table 1). Overall, a total of 14 208 participants were described across the included studies, with a total of 14 110 GBS isolates being analysed. However, this included 3752 participants and 3856 isolates originating from pregnant women, neonates (<28 days) and infants (29–90 days), which were subsequently excluded. Among non-pregnant populations, cerebrospinal fluid and blood were the main samples collected (Table S5). Most studies reported invasive GBS isolates with or without fatal outcome. Sub-group analyses provided further evidence with respect to circulating serotype and clonal complexes, as well as antibiotic resistance and virulence genes.

**Table 1.** Characteristics of included studies

Study (reference)	Country	WHO region	World Bank	Study design	Objectives	Study population [non-invasive (NI)/invasive(I)]	No. of participants*	No. of GBS
Persson, 2008 [17]	Sweden	EUR	High income	Retrospective	Investigate virulence genes in invasive GBS isolates	Neonates (I) and adults (I)	174	174
Lambertsen, 2010 [15]	Denmark	EUR	High income	Retrospective	Characterize invasive GBS isolates in adults	Adults	411	411
Meehan, 2014 [31]	Ireland	EUR	High income	Prospective	Molecular epidemiology of GBS isolates across all ages	Neonates (I), pregnant women (NI) and adults (NI/I)	177	177
Usein, 2014 [27]	Romania	EUR	Upper middle income	Prospective	Population structure of GBS isolates	Non-pregnant women (NI)	55	55
Gudjónsdóttir, 2015 [18]	Sweden	EUR	High income	Prospective	GBS serotypes	Neonates (I), infants (I) and adults (I)	317	317
Kekic, 2021 [33]	Serbia	EUR	Upper middle income	Retrospective	Maternal GBS colonization and invasive neonatal disease, and GBS serotype distribution from various patient groups	Neonates (I), pregnant (NI) and non-pregnant women (NI/I)	134	134
Kernéis, 2017 [28]	France	EUR	High income	Retrospective	GBS infections in adults	Adults (I)	163	163
Lopes, 2017 [34]	Portugal	EUR	High income	Prospective	Clonal composition and antibiotic susceptibility of adult GBS invasive disease	Adults (I)	555	555
van Kassel, 2019 [14]	Netherlands	EUR	High income	Retrospective	Serotype distribution and outcome of adult GBS meningitis	Adults (I)	33	33
Slotved, 2021 [16]	Denmark	EUR	High income	Prospective	Characterize genotypically invasive GBS isolates	Adults (I)	55	55
van Kassel, 2021 [13]	Netherlands	EUR	High income	Retrospective	Molecular epidemiology, incidence and mortality of GBS meningitis	Neonates (I), pregnant (I) and non-pregnant adults (I)	105	105
Baldan, 2021 [30]	Switzerland	EUR	High income	Prospective	Rate of vaginal colonization in women over 60 years and phenotypic and genotypic characterization	Women >60 years (NI)	259	44
Lopardo, 2003 [26]	Argentina	AMR	Upper middle income	Prospective	Serotype distribution and antibiotic susceptibility of GBS	Neonates (I) and adults (I)	31	31
Otaguiri, 2013 [25]	Brazil	AMR	Upper middle income	Prospective	Characterize GBS isolates from women of reproductive age	Non-pregnant women (NI)	83	83
Flores, 2015 [7]	USA	AMR	High income	Retrospective	Population structure of serotype V GBS in non-pregnant adults	Adults (NI)	229	229
Teatero, 2015 [8]	Canada	AMR	High income	Retrospective	Genetic diversity of invasive GBS isolates	Adults (I)	37	36
Teatero, 2014; 2015 [9, 10]	Canada	AMR	High income	Retrospective	Characterization of invasive GBS population structure and antibiotic susceptibility of serotype IV GBS isolates causing adult infections	Adults (I)	600	507^‡^
McGee [11]	USA	AMR	High income	Retrospective	Genomic sequencing and antimicrobial susceptibility testing of GBS isolates	Neonates (I), pregnant (I) and non-pregnant population (I)	6340	5834
Wang, 2014 [19]	Taiwan, ROC	WPR†	Upper middle income†	Retrospective	Investigate iGBS among non-pregnant adults	Adults (I)	345	383
Lo, 2019 [20]	Taiwan, ROC	WPR†	Upper middle income†	Prospective	Serotypes and genotypes of GBS in invasive infections in infants and children <18 years old	Neonates and children <18 years (I)	23	23
Tan, 2016 [21]	Singapore	WPR	High income	Retrospective	Characterize GBS genotypically	Adults (I)	40	36
Nagano, 2019 [22]	Japan	WPR	High income	Prospective	Genetic relatedness of penicillin-resistant GBS	Adults (I)	73	77
Zhao, 2008 [23]	Australia, New Zealand	WPR	High income	Retrospective	Provide data and inform the clinical management of GBS infections	Neonates (I), pregnant (I) and non-pregnant population (I)	408	408
Zhang Nan, 2019 [24]	PR China	WPR	Upper middle income†	Retrospective	Molecular characteristics and antibiotic susceptibility of GBS	Neonates (I), pregnant (I) and non-pregnant population (I)	17	15

*No. of participants (including pregnant women and children) among included studies and no. of GBS isolated from non-pregnant population (children >3 months, adults, elderly).

†Taiwan is considered as China in the World Bank and WHO classification. WPR, WHO Western Pacific Region; EUR, WHO European Region; AMR, WHO Americas Region; GBS, group B streptococcus; NI, non-invasive; I, infection; iGBS, invasive group B streptococcal diseases.

‡Non-pregnant population classified as children 90 days to 17 years; adults, 18 to 64 years; elderly, ≥65 years.

### Sub-group analyses

#### Capsular serotype and clonal complex

**Fig. 2.**
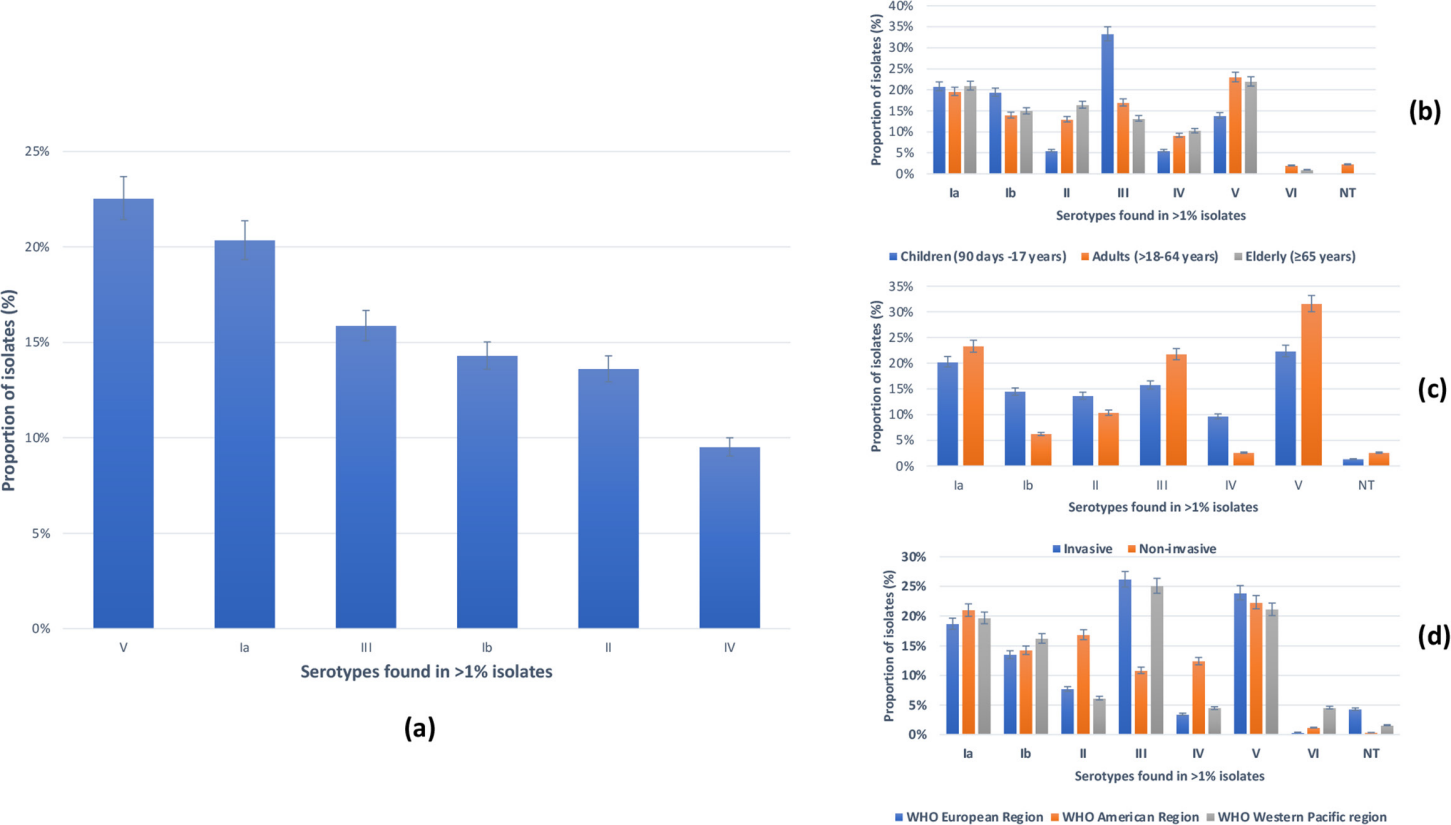
Distribution of GBS serotypes (*n*=10240) among included studies. (a) Overall distribution of GBS serotypes. (b) Sub-group analyses of GBS serotypes per age group. (c) Sub-group analyses of GBS serotypes per outcome. (d) Sub-group analyses of GBS serotypes per WHO region.

A total of 10 240 isolates from non-pregnant populations were serotyped across all included studies, with serotype V (23 %, 2310/10240) being the leading capsular serotype, followed by serotype Ia (20 %, 2084/10240) and serotype III (16 %, 1626/10240; Fig. 2a; Table S6), with strong statistical significance (*P*<0.0001). Sub-group analysis based on age groups showed that serotype III was most frequent in children (33 %, 24/72, *P*=0.004; Fig. 2b), while serotype V was most common in adults (23 %, 1622/7064, *P*<0.0001) and the elderly (21 %, 678/3104, *P*<0.0001). Serotype V was the leading serotype among invasive (22 %, 2249/10047, *P*<0.0001) and non-invasive (32 %, 61/193, *P*<0.0001) isolates with high statistical significance (Fig. 2c). In relation to serotype distribution by region, serotype V (22 %, 1520/6802) and Ia (21 %, 1429/6802) also predominated across the WHO Americas, whereas serotype III was most prevalent in the WHO European (26 %; 591/2255; *P*<0.0001) and Western Pacific (25 %; 297/1183) regions, with strong statistical significance (*P*<0.0001) (Fig. 2d).

**Fig. 3.**
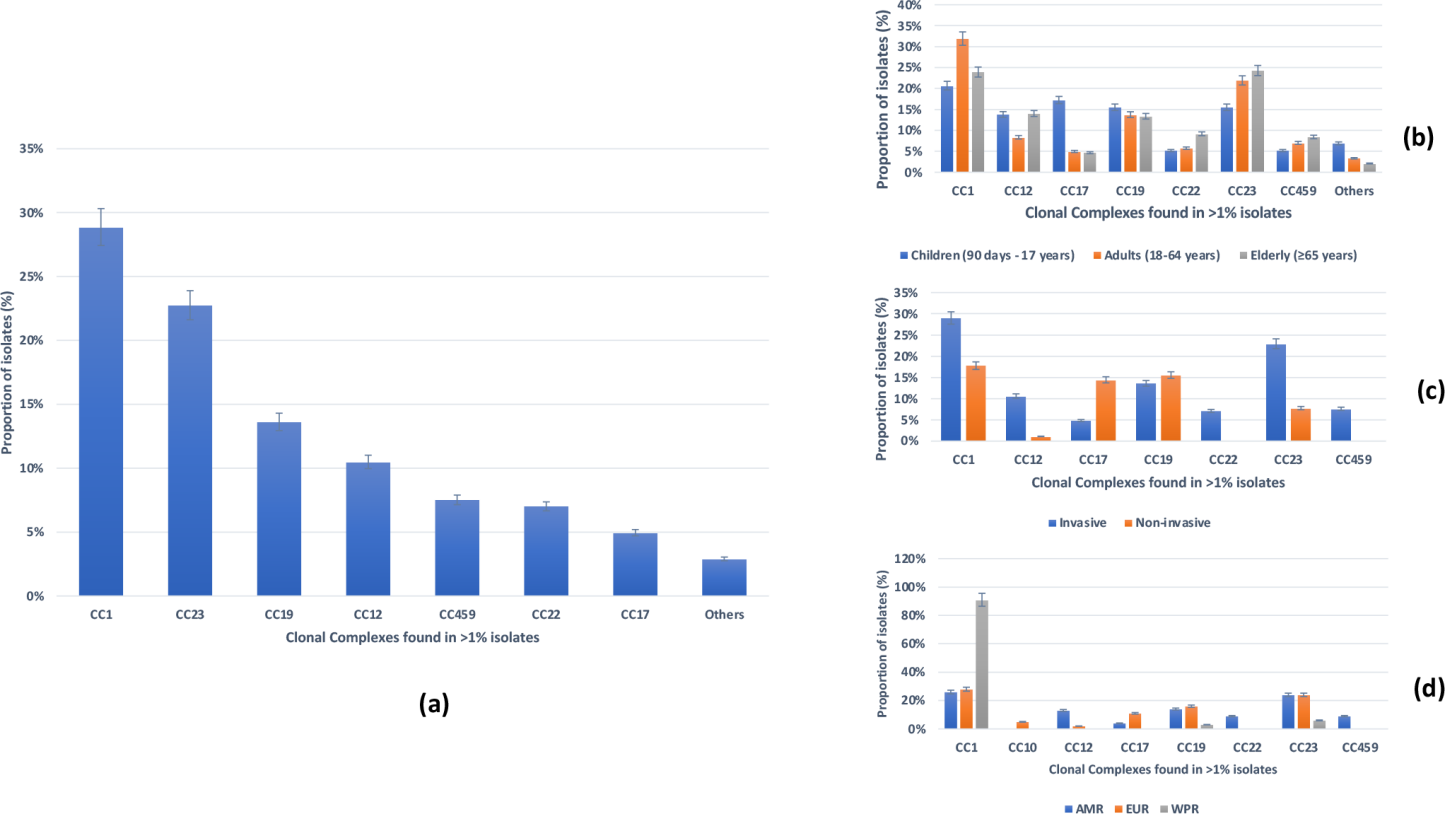
Distribution of GBS clonal complexes (*n*=7470) among included studies. (a) Overall distribution of GBS clonal complexes. (b) Sub-group analyses of GBS clonal complexes per age group. (c) Sub-group analyses of GBS clonal complexes per outcome. (d) Sub-group analyses of GBS clonal complexes per WHO region.

CCs were defined in 14 studies for 7470 isolates ([7, 9–11, 13, 14, 16, 22, 24, 27, 28, 30, 31, 34]; Fig. 3a). The leading CC detected was CC1 (29 %, 2157/7470), followed by CC23 (23 %, 1700/7470), CC19 (14 %, 1018/7470) and CC12 (10 %, 784/7470; Fig. 3a; Table S7). CC1 was the leading CC across all age groups with high statistical significance (*P*=0.0005). It was also the most frequent lineage detected in invasive (29 %, 2141/7418, *P*<0.0001) and non-invasive (31 % 16/52, *P*<0.005) disease isolates, respectively, with strong statistical significance (Fig. 3c). Finally, it predominated in the WHO Americas (26 %, 1800/6274), Western Pacific (91 %, 29/32) and European regions (28 %, 328/1164, *P*=0.0005; Fig. 3d).

#### Antibiotic resistance and surface protein genes

**Fig. 4.**
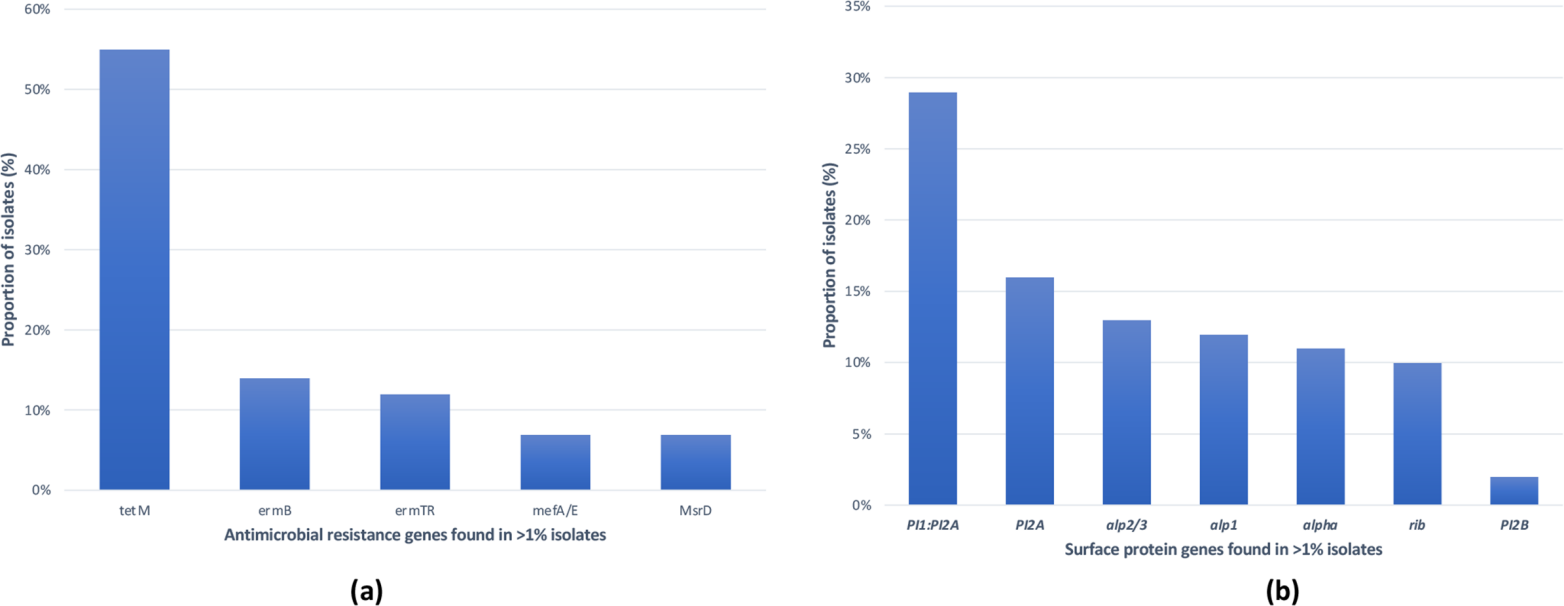
Overall distribution of antibiotic resistance (**a**) and surface protein genes (**b**).

Eleven studies reported resistance genes [7, 8, 10, 11, 17, 23, 25–27, 31, 34], while eight reported virulence factors [8, 11, 17, 22, 25, 27, 31, 34]. Among these, seven reported antibiotic resistance genes and surface protein genes concomitantly [8, 11, 17, 25, 27, 31, 34], while three described antibiotic resistance genes [7, 23, 26] only and one reported exclusively surface protein genes (22; Table S8). The *tet*M gene, encoding for tetracycline resistance, was the most common resistance gene, with a 55 % (5892/10668) prevalence (Fig. 4a). The second most common was the *erm*B gene (14 %, 1461/10668), encoding resistance to macrolides. The leading surface protein genes detected were the combination of the pilus island 1 and pilus island 2A [PI1 : PI2A (29 %, 3931/13751)], PI2A (16 %, 2180/13751) and *alp*2/3(13 %, 1745/13751) (Fig. 4b; Table S8).

### Phylogenetic analysis

Phylogenetic analysis was used to compare the relatedness of the lineages circulating among non-pregnant population in this study with the other principal lineages circulating among pregnant women and neonates. Out of 9540 GBS genomes that passed our selection criteria for this analysis, 9404 (98.5 %) passed quality control (Table S9) and comprised 6004 (63.8 %) genomes from the non-pregnant disease population, 1940 (20.6 %) genomes from infected neonates and 1414 (15 %) genomes from maternal carriage and 46 (0.6 %) from maternal disease (Table S10). The most frequent serotypes among the non-pregnant disease population were Ia (21 %, 1231/6004) and V (20 %, 1187/6004), while serotype II (24 %, 11/46) was most frequent among the maternal disease population and serotype III was dominant among invasive neonatal (59 %, 1153/1940) and maternal carriage (33 %, 472/1414) isolates, respectively (Fig. S1). ST1 was the most common ST in non-pregnant disease (22 %, 1322/6004) isolates and STs 17, 19 and 23 were most common in maternal disease (13 %, 6/46 each), whilst ST17 and ST23 predominated among neonatal invasive disease (37 %, 718/1940) and maternal carriage (18 %, 250/1414) isolates, respectively (Fig. S2).

**Fig. 5.**
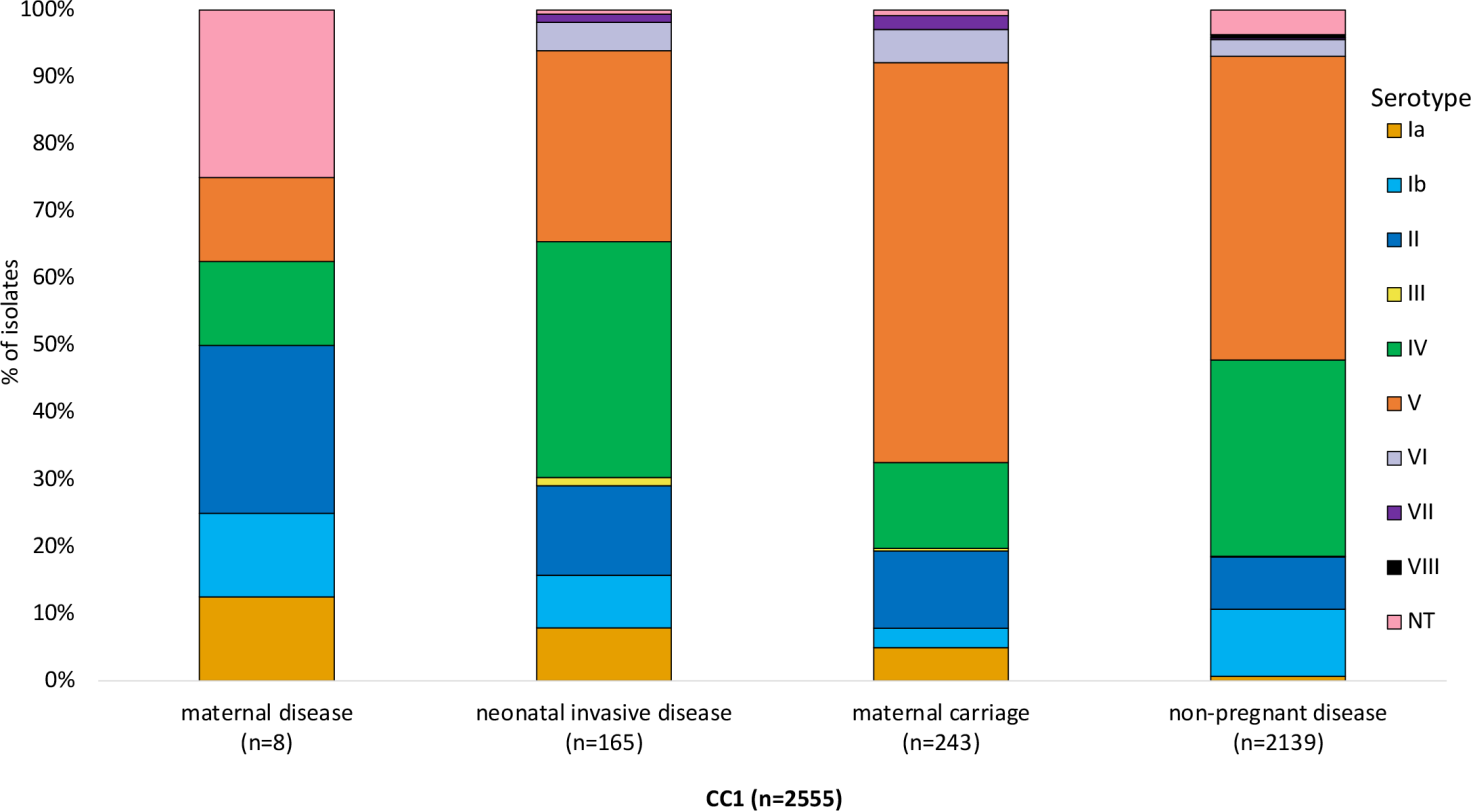
Serotype distribution of CC1 isolates (*n*=2555) per four study populations.

**Fig. 6.**
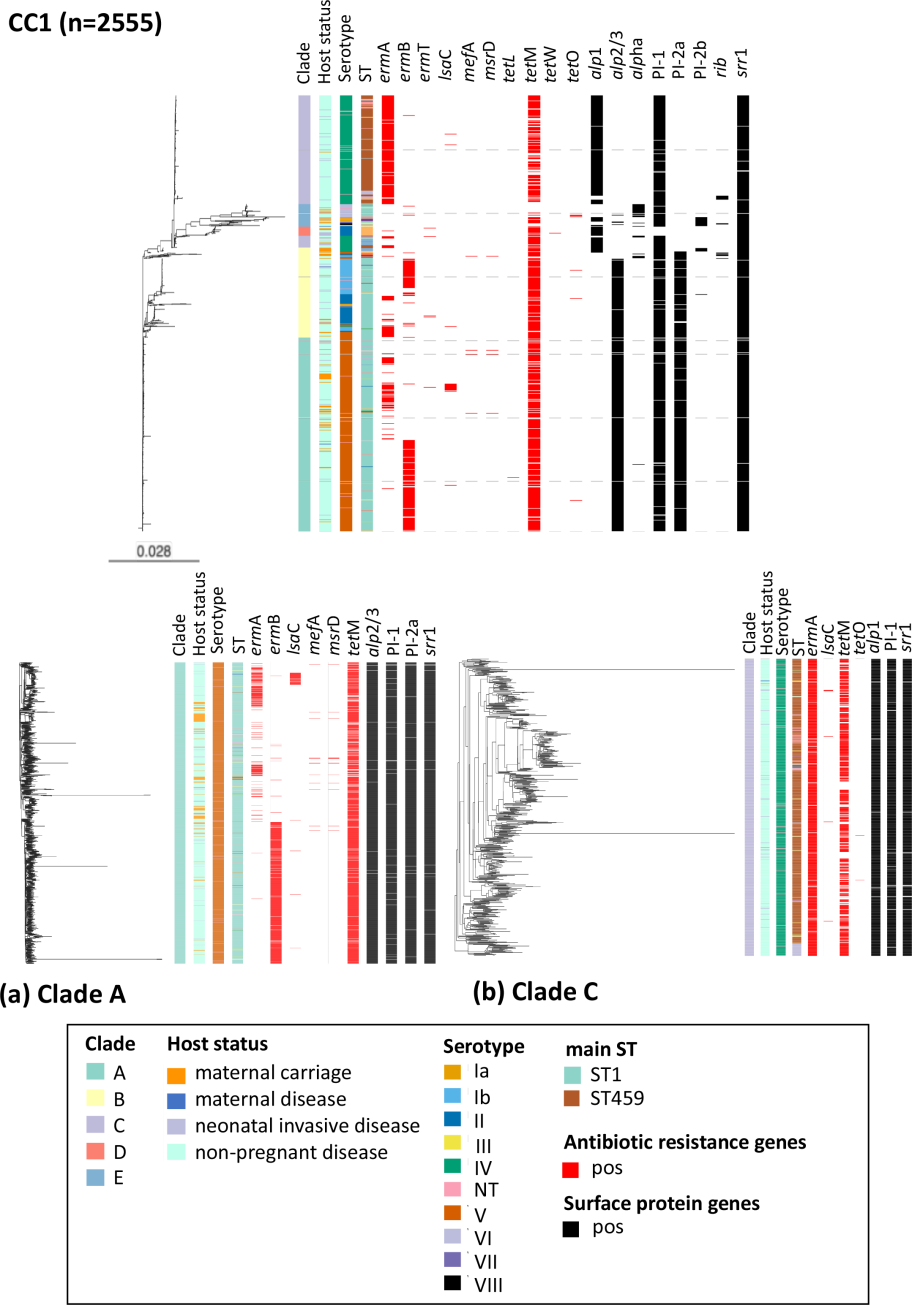
Phylogenetic relationship between GBS isolates belonging to CC1 (*n*=2555). (a) Zoom-in snapshot of clade A. (**b**) Zoom-in snapshot of clade C.

CC1 was the leading CC in the non-pregnant disease population (36 %, 2139/6004), while CC17 dominated among neonatal invasive disease (43 %, 836/1940) and maternal carriage isolates (23 %, 332/1414). Conversely, CC19 was the most prevalent CC in the isolates from maternal disease (22 %, 10/46) (Fig. S3). Among CC1 isolates, serotype V was the most dominant serotype in maternal carriage and non-pregnant disease isolates at 60 % (145/243) and 45 % (968/2139), respectively (Fig. 5). Among neonatal invasive disease CC1 isolates, serotype IV (35 %, 58/165) was the most prevalent (Fig. 5). CC1 isolates consisted of 167 different STs, with ST1 being the most common in all four study populations: maternal carriage (64 %, 156/243), non-pregnant disease (62 %, 1322/2139), maternal disease (50 %, 4/8) and neonatal invasive disease (42 %, 70/165; Fig. S4). Compared to other study populations, the highest prevalence of macrolide resistance genes in CC1 isolates was observed among non-pregnant disease isolates (84 %, 1568/2139; Table S11), most especially *erm*A (40 %, 864/2139) and *erm*B (31 %, 672/2139). Tetracycline resistance genes were prevalent in CC1 isolates (82 %, 2093/2555), with *tet*M gene being widespread (99 %, 2077/2093) in all four populations (Fig. 6). Overall, the leading surface gene profiles in CC1 were *alp*2/3+PI-1 and *srr-1* (60 %, 1544/2555) (Fig. 6). Among non-pregnant disease (64 %, 1362/2139) and maternal carriage (66 %, 161/243) isolates, the most common alpha-like protein-encoding gene was *alp*2/3, while *alp*1 was most frequent among neonatal invasive disease (54 %, 88/165) isolates (Fig. 6).

Phylogenetic analysis of CC1 revealed that isolates from all four study populations were dispersed among five CC1 clades: clade A (44 %, *n*=1133), clade C (28 %, *n*=712), clade B (21 %, *n*=527), clade E (5 %, *n*=130) and clade D (2 %, *n*=53), although clade D did not have any maternal disease isolates (Fig. S5). Most of the maternal carriage (53 %, *n*=130) and non-pregnant disease (45 %, *n*=985) isolates were represented in clade A (Figure S5). By contrast, the majority of from neonatal invasive disease isolates (33 %, *n*=54) belonged to clade C (Fig. S5). Serotype V/ST1 isolates (90 %, 1018/1133) in clade CC1-A and serotype IV/ST459 (74 %, 526/712) in clade CC1-C were the most common serotype and ST combinations (Fig. 6a, b). Among the four study populations, a high proportion of macrolide resistance genes (*erm*B and *erm*A) were found among GBS isolates from the non-pregnant population and belonging to clade CC1-A (65 %, 474/730; Figs 6a and S6) and CC1-C (64 %, 599/934; Figs 6b and S6), respectively.

## Discussion

We assessed 24 studies reporting on the molecular epidemiology of invasive and non-invasive GBS among non-pregnant populations. Our study emphasizes the significant GBS data gaps from LMICs, especially in the African and Southeast Asian regions, with none originating from these settings. It reveals that LMICs are still lagging behind high-income countries in GBS surveillance across all at-risk populations.

The study revealed that serotype V (17 %) was the leading capsular serotype detected among GBS circulating in non-pregnant populations [3]. However, a closer look at the data indicates that serotype III was essentially most prevalent in children (33 %), while serotype V was most common in adults (23 %) and the elderly (27 %). This is in contrast to the findings of two reports outlining the predominance of GBS serotype III among non-pregnant populations, neonates and pregnant women [35, 36]. Sub-group analyses also showed that GBS serotype V was the leading serotype causing invasive and non-invasive infections in non-pregnant populations across the WHO Americas region. Of note, serotype V ranks top among the five leading GBS serotypes in neonates globally [2]. Our findings highlight the importance of monitoring GBS in non-pregnant populations who could serve as a potential reservoir and source of neonatal GBS contamination.

This hypothesis is further supported by the predominance of CC1 (29 %), and particularly of CC1 causing invasive infections (29 %) in non-pregnant populations, which is in contrast to a report from neonatal/infant infections, where CC19 (22 %) and CC23 (19 %) were the predominant CCs [36]. This result suggests that sustainable containment of GBS requires that we understand the genomic features of circulating strains not only among pregnant women and children, but also among non-pregnant populations who might be involved in the evolution and subsequent dissemination of GBS.

### Antibiotic resistance genes and virulence factors

Given the increasing resistance [37] and virulence [38] of GBS isolates, it is important to monitor circulating GBS strains among non-pregnant populations. Further evidence supporting this claim is the predominance in non-pregnant populations of the surface protein genes PI1 : PI2A (29 %) and PI2A (16 %), which are associated with the ability of GBS to adhere to and invade host cells, contributing thereby to its virulence. These proteins are also present in neonatal invasive GBS isolates, some of which are targets of some vaccines currently under clinical trial [39].

On this ground, we can argue that non-pregnant populations, especially the elderly, who are particularly vulnerable to GBS, should also be considered in GBS vaccine deployment strategies, as neglecting it in this population will make the United Nations Sustainable Development Goal three of promoting health and well-being for all ages difficult to achieve [40].

Although GBS are exquisitely sensitive to penicillin, which is a first-line antibiotic for invasive GBS disease management, the emergence of reduced-susceptibility strains is of increasing concern [37]. In this study, the leading resistance genes detected among GBS isolates were the ribosomal protection protein *tetM* gene, with a prevalence of 55 %, followed by *erm*B (14 %). These genes are generally located in conjugative transposons belonging to the *Tn*916 family, where they can subsequently be transferred horizontally to other bacteria [41]. Our finding is similar to that reported in previous studies, where high rates (85–100 %) of *tet*M and *erm*B genes were recorded among GBS isolates [38, 41]. This confirms that clindamycin-resistant GBS isolates are a concerning antibiotic resistance threat [37].

Sub-analysis of CC1 revealed high macrolide resistance gene carriage in CC1 (67 %, 1703/2555), which is concordant with previous studies describing increased macrolide resistance in CC1 isolates [29]. With respect to the study population, increased macrolide resistance gene levels were observed in isolates from the non-pregnant population (84 %, 1568/2139), followed by neonatal disease isolates (44 %, 73/165), with the least prevalence observed in maternal carriage isolates (25 %, 60/243), highlighting the inadequacy of macrolides for use as an alternative for penicillin-allergic patients of all age groups. Our findings reveal that ongoing GBS prevention and control measures that mainly target pregnant women and neonates should be extended to other ecological niches, as these could serve as reservoirs and potential sources of novel GBS strains. Extensive antibiotic use in animal farming, for instance, in which GBS is also an important pathogen, could favour the emergence of increasingly resistant and/or virulent strains that could be transmitted to humans (both pregnant and non-pregnant populations). Such a scenario could thus undoubtedly compromise the efficacy of current GBS prevention strategies and policies.

### Phylogenetic analysis

Phylogenetic analysis of GBS CC1 revealed that disease isolates from the non-pregnant, neonatal and pregnant women populations interspersed with maternal carriage isolates across the CC1 tree (Fig. 6). This suggests that these isolates originated from the same genetic pool and that GBS transmission across populations is plausible. Cluster analysis showed that the majority of isolates from non-pregnant population were clustered into two major CC1 clades, CC1-A (45 %, 958/2139) and CC1-C (30 %, 647/2139). Given the association with the leading serotype and ST combinations, serotype V/ST1 isolates were dominant in CC1-A (90 %, 1018/1133) and serotype IV/ST459 isolates (74 %, 526/712) were most frequent in CC1-C (Fig. 6a, b).

The distribution of antibiotic resistance genes and surface protein genes corresponded well with the phylogenetic clusters. The macrolide resistance gene *erm*B (45 %, 515/1133) and surface proteins *alp*2/3+PI-1+PI-2a and *srr*-1 (96 %,1093/1133) were prevalent in clade CC1-A (Fig. 6a), while *erm*A (90 %, 638/712) and *alp*1+PI-1+*srr*-1 (93 %, 660/712) predominated in clade CC1-C (Fig. 6b). The clustering of *erm*B-positive isolates in clade CC1-A and *erm*A-positive isolates in clade CC1-C probably reflects an association of the *erm*B gene with serotype V and the *erm*A gene with genotype IV in the non-pregnant population.

In contrast to CC17 and CC19 isolates, in which *rib* gene is abundant (96.9 and 81.8 %, respectively) [37], in CC1 the prevalence of the *rib* gene was very low (2 %, 48/2555), with none of the CC1 isolates from maternal disease carrying the *rib* gene.

### Limitations

This systematic review has several limitations. First, the review adhered strictly to the updated PRISMA statement; however, our ability to identify all relevant studies may have been compromised by some variation in the indexing of studies in publication databases and institutional registration of authors performing the search. Second, the study populations were very diverse, with included GBS isolates originating from various sources, such as surveillance, outbreaks, and bloodstream and vaginal infections, and from both hospital- and community-acquired cases. Third, it was not possible to fully assess the genetic diversity of GBS by gender or considering co-morbidity factors. Further, the mortality rate individually or grouped by invasive and non-invasive isolates could not be ascertained. Fourth, it is a common practice to address multiple research questions in different studies with the same isolates. Whilst such isolates may have been referenced in this review, we tried our best to count them once and only where needed for each specific analysis. Fifth, we acknowledge that we did not fully capture or abstract rare STs and virulence or resistance genes that were detected in some of studies into the review dataset. Furthermore, the lack of genomic data across LMICs precludes any robust conclusion about the lineages circulating globally. Finally, some sample biases were evident from some studies with large numbers of GBS isolates with specific serotype and/or CC and genomic data originating from a only a few studies.

### Conclusion and recommendations

The study revealed that serotype V and CC1 were the leading capsular serotype and clonal complex detected among GBS circulating in non-pregnant populations, respectively. It showed that behind the tetracycline resistance gene *tet*M that is frequent in most GBS isolates, the macrolide resistance gene *ermB* displayed an elevated prevalence while the PI1 : PI2A combination comprised the leading surface protein-encoding gene. Finally, it suggested that GBS transmission across populations is plausible and can hamper ongoing prevention and containment strategies that only target pregnant women and neonates.

This study highlights and clarifies some existing knowledge gaps concerning GBS among non-pregnant population. It emphasizes that addressing data gaps is required for adequate management of this important and overlooked life-threatening pathogen. The study indicates further advanced molecular epidemiology studies, especially in LMICs, for evidence-based decisions and sustainable containment of GBS.

## Supplementary Data

Supplementary material 1
