# Supplementary material for: Molecular epidemiology of Streptococcus agalactiae in non-pregnant populations: a systematic review
Source: Microb Genom. 2023 Nov 29;9(11):001140. doi: 10.1099/mgen.0.001140 (PMC10711312; doi:10.1099/mgen.0.001140)
Supplement: Supplementary material 1 [file mgen-9-1140-s001.pdf]

Molecular Epidemiology of *Streptococcus agalactiae* in non-pregnant populations: A Systematic Review

Luria Leslie Founou[1,2,3*†], Uzma Basit Khan[4†], Nubwa Medugu[5], Tatiana C.A. Pinto[6], Saffiatou Darboe[7], Zhu Chendi[8], Raspail Carrel Founou[3,9,10], Ka-Ning To[11], Dorota Jamrozy[4], Konstantinos Karampatsas[11], Victoria R. Carr[4,12], Kevin Pepper[4], Ziyaad Dangor[13], Margaret Ip[8], Kirsty Le Doare[14], Stephen D. Bentley[4,15#]

Supplementary materials

Table S1. PRISMA checklist

Section and Topic	Item #	Checklist item	Location where item is reported
**TITLE**			
Title	1	Identify the report as a systematic review.	P1
**ABSTRACT**			
Abstract	2	See the PRISMA 2020 for Abstracts checklist.	P2
**INTRODUCTION**			
Rationale	3	Describe the rationale for the review in the context of existing knowledge.	p3
Objectives	4	Provide an explicit statement of the objective(s) or question(s) the review addresses.	p3
**METHODS**			
Eligibility criteria	5	Specify the inclusion and exclusion criteria for the review and how studies were grouped for the syntheses.	P4, Table S3
Information sources	6	Specify all databases, registers, websites, organisations, reference lists and other sources searched or consulted to identify studies. Specify the date when each source was last searched or consulted.	P4, Table S2
Search strategy	7	Present the full search strategies for all databases, registers and websites, including any filters and limits used.	P4, Table S2
Selection process	8	Specify the methods used to decide whether a study met the inclusion criteria of the review, including how many reviewers screened each record and each report retrieved, whether they worked independently, and if applicable, details of automation tools used in the process.	P4-5, Table S3
Data collection process	9	Specify the methods used to collect data from reports, including how many reviewers collected data from each report, whether they worked independently, any processes for obtaining or confirming data from study investigators, and if applicable, details of automation tools used in the process.	P4-5
Data items	10a	List and define all outcomes for which data were sought. Specify whether all results that were compatible with each outcome domain in each study were sought (e.g. for all measures, time points, analyses), and if not, the methods used to decide which results to collect.	P3-5, Table S5
	10b	List and define all other variables for which data were sought (e.g. participant and intervention characteristics, funding sources). Describe any assumptions made about any missing or unclear information.	P3-5, Table S5
Study risk of bias assessment	11	Specify the methods used to assess risk of bias in the included studies, including details of the tool(s) used, how many reviewers assessed each study and whether they worked independently, and if applicable, details of automation tools used in the process.	P5, Table S4
Effect measures	12	Specify for each outcome the effect measure(s) (e.g. risk ratio, mean difference) used in the synthesis or presentation of results.	NA
Synthesis	13a	Describe the processes used to decide which studies were eligible for each synthesis (e.g. tabulating the study intervention characteristics and	P4

Section and Topic	Item #	Checklist item	Location where item is reported
methods		comparing against the planned groups for each synthesis (item #5)).	
	13b	Describe any methods required to prepare the data for presentation or synthesis, such as handling of missing summary statistics, or data conversions.	P4-5
	13c	Describe any methods used to tabulate or visually display results of individual studies and syntheses.	P4-5
	13d	Describe any methods used to synthesize results and provide a rationale for the choice(s). If meta-analysis was performed, describe the model(s), method(s) to identify the presence and extent of statistical heterogeneity, and software package(s) used.	NA
	13e	Describe any methods used to explore possible causes of heterogeneity among study results (e.g. subgroup analysis, meta-regression).	NA
	13f	Describe any sensitivity analyses conducted to assess robustness of the synthesized results.	NA
Reporting bias assessment	14	Describe any methods used to assess risk of bias due to missing results in a synthesis (arising from reporting biases).	P4
Certainty assessment	15	Describe any methods used to assess certainty (or confidence) in the body of evidence for an outcome.	P4
**RESULTS**			
Study selection	16a	Describe the results of the search and selection process, from the number of records identified in the search to the number of studies included in the review, ideally using a flow diagram.	P7-8, Figure 1
	16b	Cite studies that might appear to meet the inclusion criteria, but which were excluded, and explain why they were excluded.	P7-8, Figure 1
Study characteristics	17	Cite each included study and present its characteristics.	P7-8, Table 1, Table S5
Risk of bias in studies	18	Present assessments of risk of bias for each included study.	P7-8, Table S4
Results of individual studies	19	For all outcomes, present, for each study: (a) summary statistics for each group (where appropriate) and (b) an effect estimate and its precision (e.g. confidence/credible interval), ideally using structured tables or plots.	Table 1, Table S5
Results of syntheses	20a	For each synthesis, briefly summarise the characteristics and risk of bias among contributing studies.	P7-11
	20b	Present results of all statistical syntheses conducted. If meta-analysis was done, present for each the summary estimate and its precision (e.g. confidence/credible interval) and measures of statistical heterogeneity. If comparing groups, describe the direction of the effect.	P7-11
	20c	Present results of all investigations of possible causes of heterogeneity among study results.	P7-11
	20d	Present results of all sensitivity analyses conducted to assess the robustness of the synthesized results.	P7-11
Reporting biases	21	Present assessments of risk of bias due to missing results (arising from reporting biases) for each synthesis assessed.	P7-11
Certainty of evidence	22	Present assessments of certainty (or confidence) in the body of evidence for each outcome assessed.	P7-11
**DISCUSSION**			
Discussion	23a	Provide a general interpretation of the results in the context of other evidence.	P12-15
	23b	Discuss any limitations of the evidence included in the review.	P12-14

Section and Topic	Item #	Checklist item	Location where item is reported
	23c	Discuss any limitations of the review processes used.	P12-14
	23d	Discuss implications of the results for practice, policy, and future research.	P12-14
**OTHER INFORMATION**			
Registration and protocol	24a	Provide registration information for the review, including register name and registration number, or state that the review was not registered.	P4
	24b	Indicate where the review protocol can be accessed, or state that a protocol was not prepared.	P4
	24c	Describe and explain any amendments to information provided at registration or in the protocol.	NA
Support	25	Describe sources of financial or non-financial support for the review, and the role of the funders or sponsors in the review.	P15
Competing interests	26	Declare any competing interests of review authors.	P14
Availability of data, code and other materials	27	Report which of the following are publicly available and where they can be found: template data collection forms; data extracted from included studies; data used for all analyses; analytic code; any other materials used in the review.	P14

From: Page MJ, McKenzie JE, Bossuyt PM, Boutron I, Hoffmann TC, Mulrow CD, et al. The PRISMA 2020 statement: an updated guideline for reporting systematic reviews. BMJ 2021;372:n71. doi: 10.1136/bmj.n71

For more information, visit: http://www.prisma-statement.org/

Table S2: Search strategy

**Date of search performed**	20th September 2021
**Databases searched**	PubMed, Cochrane Library, ISI Web of Knowledge, EBSCOhost, Chinese database of the World Health Organization Library Information System (WHOLIS), Literature in Health Sciences in Latin America and the Caribbean (LILACS), China National Knowledge Infrastructure database, Wanfang med online database, African Journals Online
**Search terms**	Molecular epidemiology OR genetic diversity OR mechanisms of resistance OR drug resistance OR antibiotic resistance gene OR antimicrobial resistance OR genotyping OR serotyping OR AMR genotype<br>AND<br>Streptococcus agalactiae OR group B streptococcus OR Group B streptococcal OR Group B streptococcal infection OR Group B streptococcal colonization OR Group B streptococcal colonisation<br>AND<br>Male OR men OR infants OR child* OR women OR female OR non-pregnant OR elderly OR aged<br>NOT<br>pregnant women OR review OR conference OR letter OR correspondence
**Restrictions**	01 January 2000 onwards

Table S3: Inclusion and Exclusion Criteria

**Inclusion criteria**
- Original research<br>- Minimum 20 patients or 20 GBS isolates<br>- Studies reporting at least serotype of GBS in neonates > 3 months, and/or non-pregnant adults (men, women, elderly) and,<br>- Studies reporting on molecular characteristics of GBS i.e serotype and/or, sequence type, and/or specific surface protein genes, and/or antimicrobial resistance genes, and/or virulence genes and/or mobile genetic elements of isolated GBS in the selected populations
**Exclusion criteria**
- Studies dealing only with pregnant women and neonates < 3 months<br>- Studies dealing only with prevalence and AST of GBS in pregnant and/or non-pregnant population<br>- Studies published prior 2000<br>- Non-published papers,<br>- Review, letter to editor, conference paper, case report,<br>- Studies with less than 20 participants or 20 GBS isolates

Table S4. Quality Assessment Sheet

Author names	Publication Year	1.Was the sample frame appropriate to address the target population?	2.Were study participants recruited in an appropriate way?	3.Was the sample size adequate?	4. Were the study subjects and setting described in detail?	5. Was data analysis conducted with sufficient coverage of the identified sample?	6. Were valid methods used for the identification of the condition?	7. Was the condition measured in a standard, reliable way for all participants?	8. Was there appropriate statistical analysis?	9. Was the response rate adequate, and if 0t, was the low response rate managed appropriately?	Score	Status >6: High, 3-6: Moderate, <3: poor
**Gudjónsdóttir**	2015	1	1	1	0	1	1	1	1	1	8	High
**Kekic**	2021	1	0	1	1	1	1	1	1	1	8	High
**Kernéis**	2017	1	1	1	1	1	1	1	1	1	9	High
**Lambertsen**	2010	1	1	1	0	1	1	1	1	1	8	High
**Lo**	2019	1	1	1	1	1	1	1	1	0	8	High
**Lopardo**	2003	1	1	0	0	0	1	1	0	1	5	Moderate
**Lopes**	2017	1	1	1	0	1	1	1	1	0	7	High
**Meehan**	2014	1	1	1	0	1	1	1	1	0	7	High
**Nagano**	2019	1	0	1	0	1	1	1	0	0	5	Moderate
**Otaguiri**	2013	1	0	1	1	1	1	1	1	0	7	High
**Persson**	2008	1	0	1	1	1	1	1	1	0	7	High
**Slotved**	2021	1	0	1	1	1	1	1	1	0	7	High
**Teatero**	2015a	1	0	1	0	0	1	1	1	0	5	Moderate
**Teatero**	2014 ;2015b	1	1	1	0	1	1	1	1	1	8	High
**Usein**	2014	1	0	1	0	1	1	1	1	0	6	Moderate
**Tan**	2016	1	0	1	0	0	1	1	1	1	6	Moderate
**van Kassel,**	2019	1	1	1	1	1	1	1	1	1	9	High
**van Kassel,**	2021	1	1	1	1	1	1	1	1	1	9	High
**Wang**	2014	1	1	1	1	1	1	1	1	1	9	High
**Zhao**	2008	1	1	1	1	1	1	1	1	1	9	High
**Zhang Nan**	2019	1	1	0	1	1	1	1	1	1	8	High
**Baldan**	2021	1	1	1	0	1	1	1	1	1	8	High
**Flores**	2015	1	1	1	0	1	1	1	1	1	8	High

Table S5. Overall distribution of sample type from included articles

Author names	Publication Year	Blood	CSF	Joint fluids or other sterile site	Urine	Blood and CSF	Vaginal/rectal swab	Tissue	Total No sample
Gudjónsdóttir et al.	2015	na	na	na	na	na	na	na	410*
Kekic et al.	2021	68	8	4	20	0	971	0	1071
Kernéis et al.	2017	17	1	120	4	26	0	0	168
Lambertsen et al.	2010	na	na	na	na	na	na	na	411*
Lo et al.	2019	146	6	0	0	25	0	0	177
Lopardo et al.	2003	23	2	4	0	3	0	12	44
Lopes et al.	2017	468	11	76	0	0	0	0	555
Meehan et al.	2014	na	na	na	na	na	na	na	177*
Nagano et al.	2019	4	0	70	3	0	0	0	74
Otaguiri et al.	2013	0	0	0	52	0	31	0	52
Persson et al.	2008	0	0	297	0	0	0	0	297
Slotved et al.	2021	0	0	55	0	0	0	0	55
Tan et al.	2016	98	0	0	0	0	0	0	98
Usein et al.	2014	0	0	0	0	0	55	0	55
van Kassel et al.	2019	0	2579	0	0	0	0	0	2579
van Kassel et al.	2021	875	228	0	0	398	0	0	1501
Wang et al.	2014	na	na	na	na	na	na	na	383*
Zhao et al.	2008	605	35	23	0	0	0	0	663
Zhang	2019	2	0	2	8	na	3	0	15
Baldan et al.	2021	0	0	0	0	0	255	0	255
McGee et al.	2021	5503	72	767	0	0	0	0	6342
Teatero et al.	2015a	47	0	20	0	0	0	18	85
Teatero et al.	2014 ; 2015b	430	2	60	0	0	0	15	507

*Not available: Number of sample positive to GBS were not specified in the study.

Table S6. Overall distribution of GBS serotype from included articles

Author names	Publication Year	Serotype											Grand Total
		Ia	Ib	II	III	IV	V	VI	VII	VIII	IX	NT	
Gudjónsdóttir	2015	34	45	29	62	14	124	0	0	3	7	0	318
Kekic	2021	15	17	13	32	9	37	0	0	0	0	0	123
Kernéis	2017	26	12	19	47	12	40	3	3	0	0	1	163
Lambertsen	2010	81	38	23	121	18	77	0	0	7	1	45	411
Lo	2019	3	3	0	12	0	1	4	0	0	0	1	24
Lopardo	2003	9	1	8	5	5	0	0	0	0	0	3	31
Lopes	2017	169	133	37	70	9	102	2	0	2	8	23	555
Meehan	2014	4	3	8	3	3	8	1	0	0	1	0	31
Nagano	2019	46	3	0	28	0	0	0	0	0	0	1	78
Otaguiri	2013	35	0	9	12	0	25	0	0	0	1	1	83
Persson	2008	14	24	17	47	11	58	0	0	0	0	3	174
Slotved	2021	19	7	4	3	3	14	1	2	2	0	0	55
Tan	2016	1	0	4	26	0	0	4	1	0	0	0	36
Usein	2014	3	2	9	18	3	20	0	0	0	0	0	55
van Kassel	2019	8	2	1	13	2	4	1	1	/	/	0	32
van Kassel	2021	23	12	3	42	2	7	1	/	/	/	5	95
Wang	2014	19	91	15	58	33	113	36	4	/	/	14	383
Zhao	2008	92	54	37	95	16	115	11	3	2	/	/	425
Zhang	2019	1	8	0	0	0	5	0	0	0	0	0	14
Baldan	2021	6	2	2	12	2	11	1	0	0	0	0	36
Flores	2015	0	0	0	0	0	229	0	0	0	0	0	229
McGee	2021	1300	875	1037	735	704	1081	59	5	17	8	13	5834
Teatero	2015a	71	79	61	75	93	112	15	3	15	2	23	549
Teatero et al.	2014 ;2015b	105	54	58	110	36	127	5	1	1	2	7	506
**Grand Total**		**2084**	**1465**	**1394**	**1626**	**975**	**2310**	**144**	**23**	**49**	**30**	**140**	**10240**

Table S7. Overall distribution of GBS Clonal complexes from included articles

Author names	Publication Year	Clonal Complexes																				
		CC1	CC4	CC6	CC7	CC8	CC17	CC10	CC12	CC19	CC22	CC23	CC24	CC26	CC28	CC49	CC103	CC297	CC130	CC196	CC459	other CC#
Kernéis	2017	24	1	/	4	2	17	8	1	5	0	17	0	0	0	0	0	0	0	0	0	5[&]
Lopes	2017	224	6	0	1	0	32	37	0	73	0	157	0	7	0	0	2	0	8	0	0	2[$]
Meehan	2014	12	0	0	0	0	1	0	8	3	0	5	0	0	0	0	0	0	0	0	0	2**
Nagano	2019	31	0	0	0	0	0	0	0	0	0	0	0	0	0	0	0	0	0	0	0	0
Slotved	2021	14	0	9	0	9	0	9	0	4	0	4	0	0	0	0	0	0	3	2	0	0
Usein	2014	8	0	0	0	0	13	1	1	9	0	3	0	1	6	1	1	2	0	0	0	6
van Kassel,	2019	5	0	0	0	0	8	3	0	4	0	9	0	0	0	0	0	0	0	0	0	3
van Kassel,	2021	14	0	0	0	0	9	0	0	66	0	44	13	0	0	0	0	0	0	0	0	0
Zhang	2019	4	0	0	0	0	0	0	0	1	0	2	0	0	0	0	0	0	0	0	0	8
Baldan	2021	4	0	0	0	0	0	0	0	4	0	2	0	0	0	0	0	2	0	0	0	19
Flores	2015	210	0	0	0	0	0	0	0	8	0	0	0	0	0	0	0	0	0	0	0	11
McGee	2021	1363	0	0	0	0	252	0	774	841	524	1445	0	0	0	0	0	0	0	0	562	70*
Teatero	2015a	243	/	/	/	/	/	/	/	/	/	12	/	/	/	/	/	/	/	/	/	/
Teatero et al.	2014 ; 2015b	1	0	0	0	0	38	0	0	0	0	0	0	0	0	0	0	0	0	0	0	91
**Grand Total (n=7470)**		**2157**	**7**	**9**	**5**	**11**	**370**	**58**	**784**	**1018**	**524**	**1700**	**13**	**8**	**6**	**1**	**3**	**4**	**11**	**2**	**562**	**217**

[&]Other CC include CC27, 167, 186, 238; $Other CC include CC773, 775; ** Other include 2 singleton not specify, * Other CC include Singleton not reported, CC585/41, 26/1087, 328, 3; #Other CCs including CC3, CC9, CC24, CC26, CC28, CC49, CC103, CC297, CC130, CC196, CC328, CC26/1087, CC585/41

Table S8. Summary of resistance genes and virulence factors detected in GBS from included articles.

Author names	Publication Year	Resistance genes												
		NO ARGs	tetM	tetO	ermA	ermB	ermTR	mefA/E	aphA-3	aad-6	tetM/tetO	tetO/tetL	MsrD	Total
Lopardo	2003	0	36	3	2	0	1	2	0	0	2	0	0	46
Lopes	2017	0	457	4	0	148	33	2	0	0	14	1	0	659
Meehan	2014	0	0	0	0	16	11	6	0	0	0	0	0	33
Otaguiri	2013	0	0	0	9	4	0	5	0	0	0	0	0	18
Persson	2008	/	/	/	/	4	/	/	/	/	/	/	0	4
Usein	2014	/	35	8	10	9	0	4	0	0	0	0	0	66
Zhao	2008	73	560	13	17	22	0	15	8	11	0	0	0	719
Flores	2015	/	183	0	0	78	41	0	0	0	0	0	0	302
McGee	2021	/	4597	221	0	1180	1139	704	32	0	83	0	728	8684
Teatero	2015a	/	/	/	/	/	93	/	/	/	/	/	0	93
Teatero et al.	2014 ; 2015b	/	24	/	19	/	1	/	/	/	/	/	/	44
**Grand Total**		**73**	**5916**	**249**	**76**	**1461**	**1320**	**738**	**40**	**11**	**99**	**1**	**728**	**10668***

*Total number of resistance genes detected among all GBS isolates

Author names	Publication Year	Virulence genes															Grand Total
		bca	alp1	alp2/3	rib	eps	bac	cylE	hylB	hvgA	alpha	PI1	PI1:PI2A	PI1:PI2B	PI2A	PI2B	
Lopes	2017	115	0	210	117	113	0	0	0	/	0	0	333	0	177	9	1074
Meehan	2014	31	0	31	70	42	26	0	0	/	0	135	0	0	126	50	511
Nagano	2019	0	0	0	0	0	0	0	0	/	0	0	0	0	0	0	0
Otaguiri	2013	0	0	0	0	0	0	83	83	/	0	0	42	4	30	7	249
Persson	2008	10	20	60	52	/	33	/	/	/	0	0	0	0	0	0	175
Usein	2014	5	12	7	18	/	1	/	/	/	0	3	29	8	8	7	98
McGee	2021	/	1658	1437	1122	/	/	/	/	/	1567	11	3527	283	1839	162	11606
Teatero et al.	2014 ; 2015b	/	/	/	/	/	/	/	/	38	0	0	0	0	0	0	38
**Grand Total**		**161**	**1690**	**1745**	**1379**	**155**	**60**	**83**	**83**	**38**	**1567**	**149**	**3931**	**295**	**2180**	**235**	**13751#**

#Total number of virulence genes detected among all GBS isolates

Table S9. Description of 9404 GBS isolates used in this study.

Download the table from https://github.com/UzmaBasit/GBS_systematic_review_non-pregnant_population/tree/main

Table S10. Country wise distribution of 9404 GBS isolates by study populations.

Country	Neonatal invasive disease	Non-pregnant disease	Maternal disease	Maternal carriage	Grand Total
Angola	14	/	/	/	14
Canada	82	221	/	/	303
Denmark	/	53	/	/	53
France	19	/	/	/	19
Kenya	/	/	/	890	890
Portugal	/	2	/	/	2
The Netherlands	1339	/	/	/	1339
UK	/	/	/	524	524
USA	486	5728	46	/	6260
**Grand Total**	**1940**	**6004**	**46**	**1414**	**9404**

Table S11. Percentage of CC1 isolates (n=2555) by GBS populations that harboured at least one macrolide resistance gene (*erm*A, *erm*B, *erm*T, *lsa*C, *mef*A and *msr*D).

GBS population	Isolate with at least one macrolide resistance gene	% of isolates
Maternal carriage (n=243)	60	25%
Maternal disease (n=8)	3	38%
Neonatal invasive disease (n=165)	73	44%
Non-pregnant disease (n=2139)	1568	84%

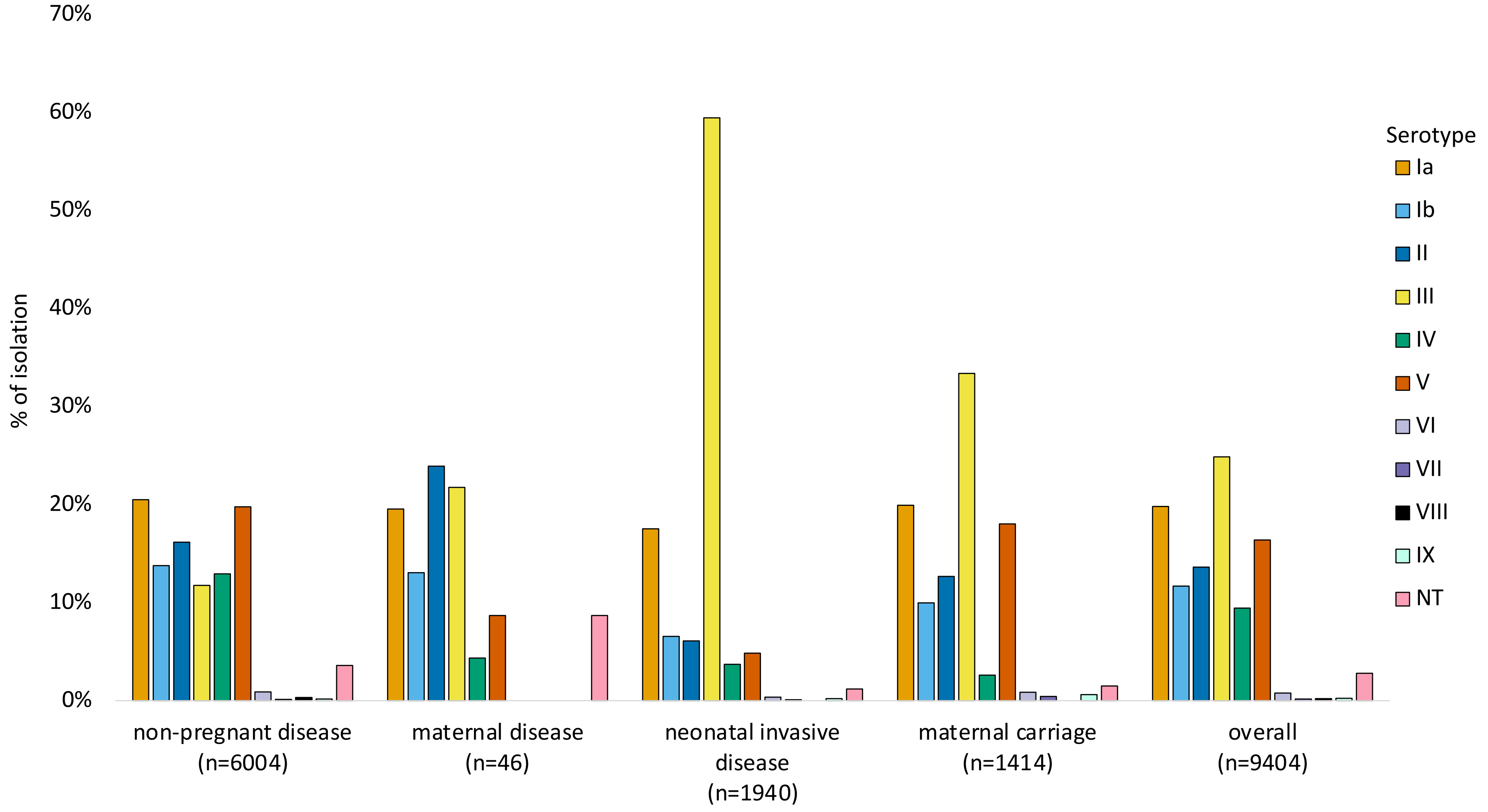

Figure S1. Serotype distribution of 9404 GBS by four study populations.

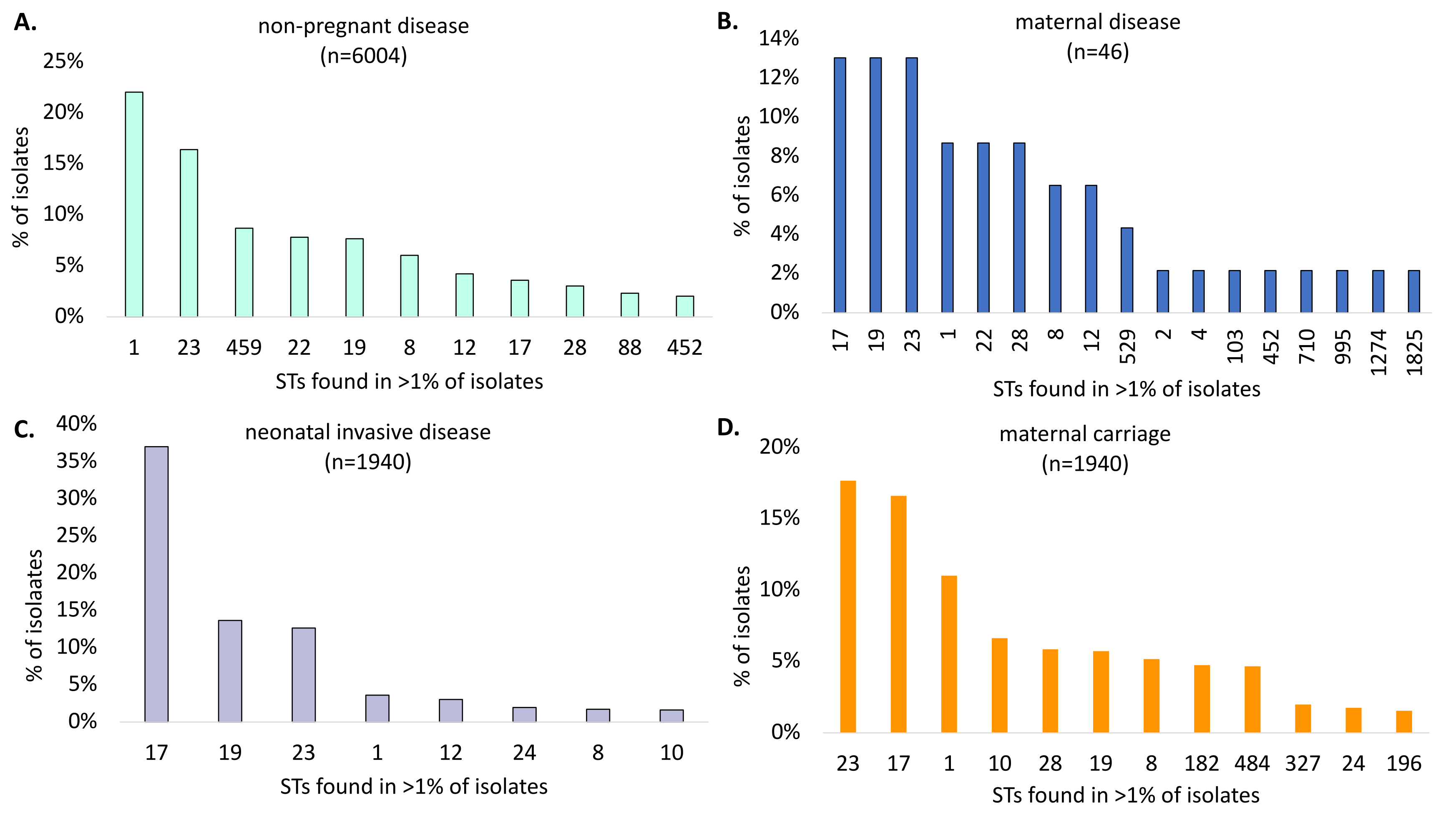

Figure S2. Commons STs observed in 9404 GBS isolates by different populations.

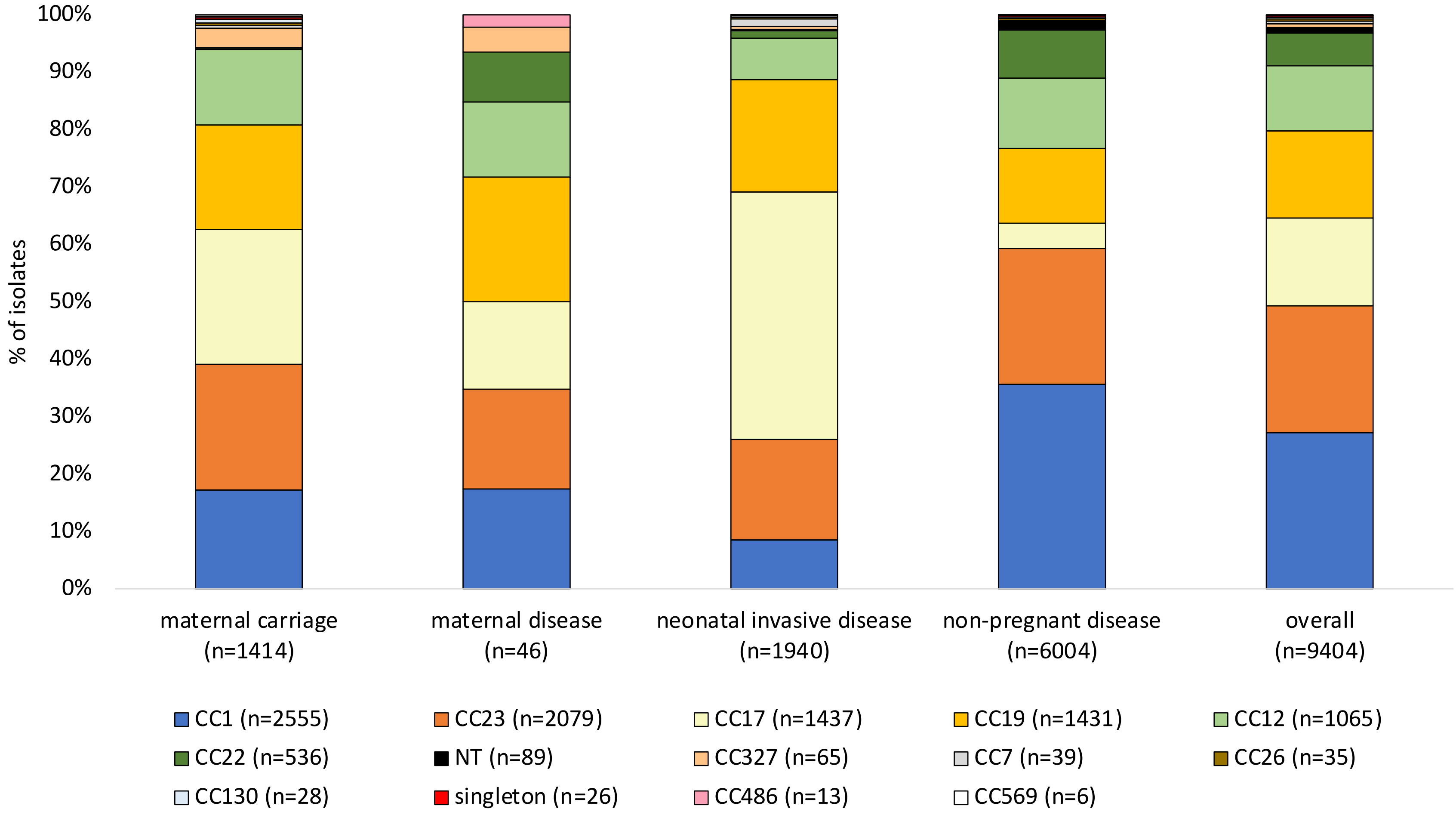

Figure S3. Distribution of CCs in 9404 GBS by study populations.

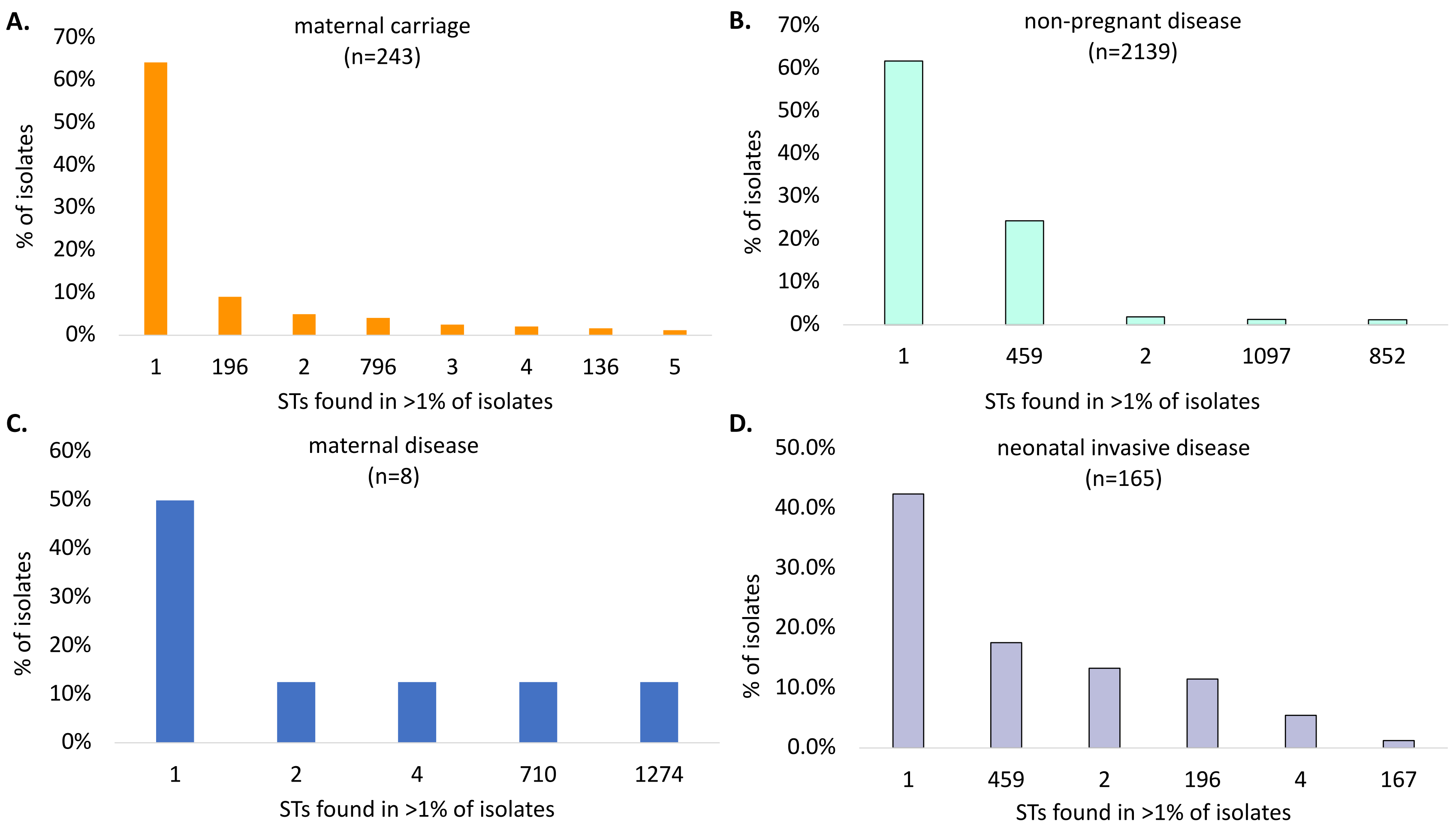

Figure S4. Commons STs observed in CC1 isolates (n=2555) by study populations.

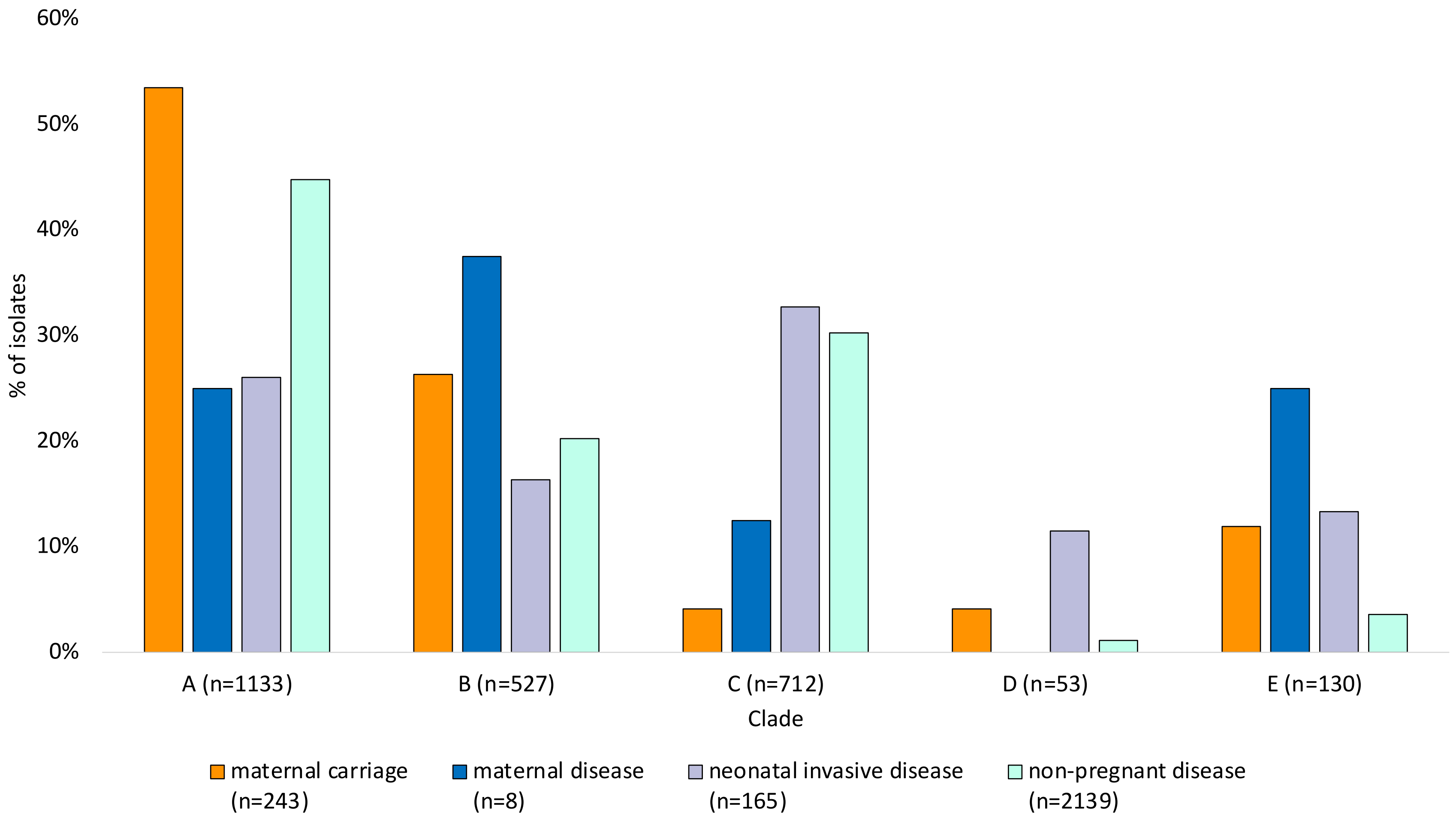

Figure S5: The distribution of isolates by study populations in CC1 clades (A-E).

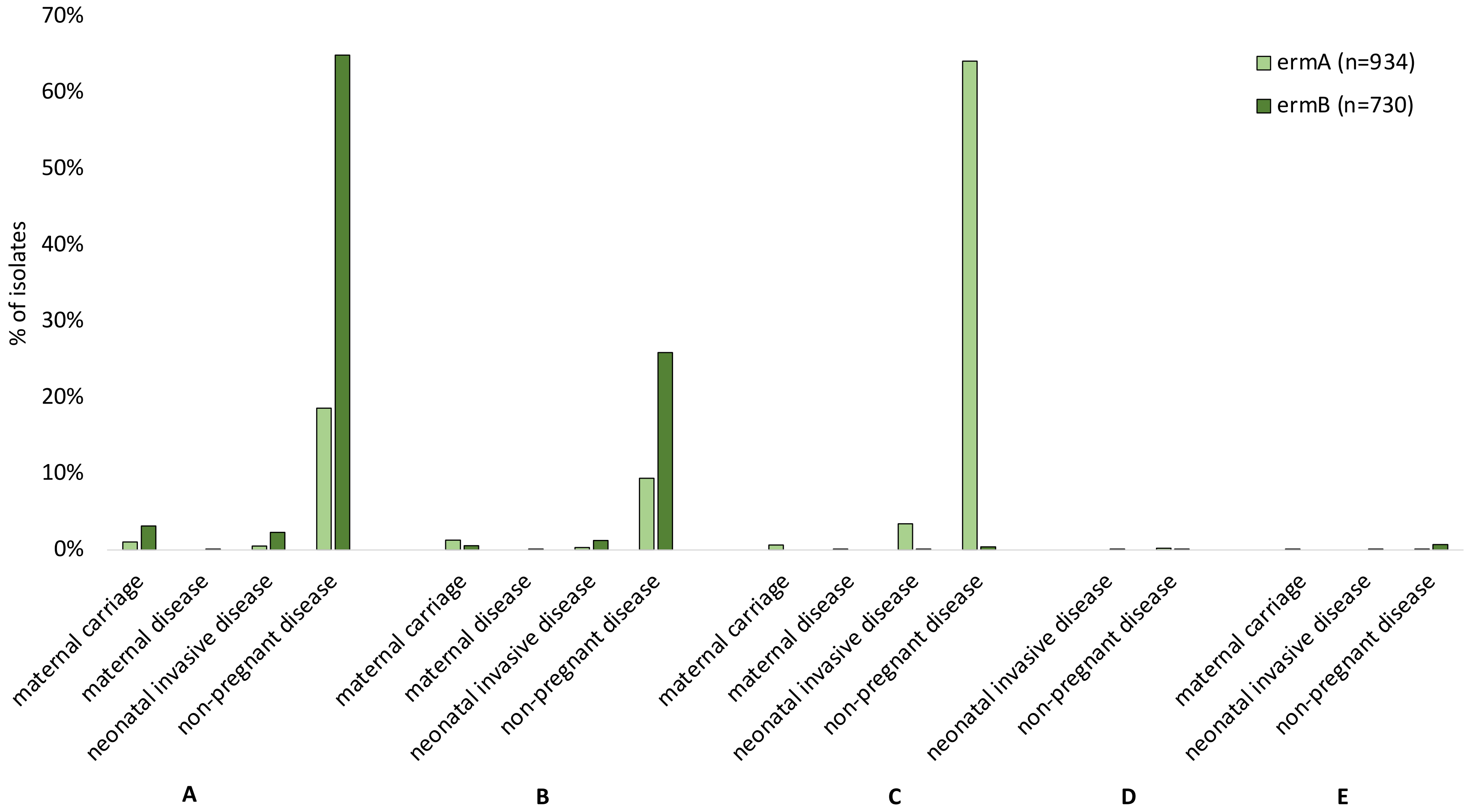

Figure S6. Distribution of macrolide resistance genes (*erm*A and *erm*B) in CC1 clades per study populations.
